# Predictors associated with CD4 cell count changes over time among HIV-infected children on anti-retroviral therapy follow-up in Mekelle General Hospital, Northern Ethiopia, 2019: a retrospective longitudinal study

**DOI:** 10.1186/s12887-023-04401-7

**Published:** 2023-12-12

**Authors:** Gebru Gebremeskel Gebrerufael

**Affiliations:** Department of Statistics, College of Natural and Computational Science, Adigrat University, P.O. Box 50, Adigrat, Ethiopia

**Keywords:** CD4 cell count, ART, Children, S-I linear mixed model, Predictors

## Abstract

**Introduction:**

AIDS continues to be a serious global public health issue. It targets CD4 cells and immunological cells, which are in charge of the body's resistance against pathogenic pathogens. In situations with limited resources, CD4 cell measurement is essential for assessing treatment responses and clinical judgments in HIV-infected children receiving Anti-Retroviral Therapy (ART). The volatility of CD4 cells during ART follow-up is still largely uncharacterized, and there are few new datasets on CD4 cell changes over time. Therefore, the purpose of this analysis was to identify the factors that were predictive of CD4 cell count changes over time in children who started ART at Mekelle General Hospital in northern Ethiopia.

**Methods:**

A retrospective follow-up study was done. 437 patients in Mekelle general hospital, northern Ethiopia, from 2014–2016 were involved. All patients who have started anti-retrieval treatment (ART) and measured their CD4 cell count at least twice, including the baseline and those who initiated ART treatment, were included in the study population. An exploratory data analysis and linear mixed model analysis were used to explore the predictors of CD4 cell count change in patients and consider variability within and between patients.

**Results:**

This study found the correlation variation explained in cells accounted for between patients was 61.3%, and the remaining 38.7% variation existed within. This indicates that there is a substantial change in random slope and intercept between and within patients. WHO clinical stage IV (β = -1.30, 95% CI: -2.37, -0.23), co-infection HIV/TB (β = -1.78, 95% CI: -2.58, -0.98), children aged 2–5 (β = -0.43; 95% CI: -0.82, -0.04), and 6–14 years (β = -1.02; 95% CI: -1.47, -0.56), non-opportunistic infection (β = 1.33, 95% CI: 0.51, 2.14), and bedridden functional status (β = -1.74, 95% CI: -2.81, -0.68) were predictors of cell changes over time.

**Conclusions:**

This study found that patients receiving ART experienced a significant change in CD4 cells over time. Because 61.3% of the variation in CD4 cells explained between patients and the remaining 38.7% within patients, such nested data structures are often strong correlation evidence. Co-infection of HIV/TB, functional status, age category of children, WHO clinical stage, and opportunistic infections are potential predictors of CD4 cells count change.

Hence, special guidance and attention is also required, especially for those patients who have an opportunistic infections, higher WHO clinical stages, co-infections with HIV and TB, and bedridden functional status.

**Supplementary Information:**

The online version contains supplementary material available at 10.1186/s12887-023-04401-7.

## Introduction

Acquired Immune Deficiency Syndrome (AIDS) is caused by the Human Immunodeficiency Virus (HIV), which decreases a person’s ability to fight infection by reducing CD4 cell count and attacking an immune cell that is responsible for the body’s immune response to infectious agents [1–3]. HIV/AIDS remains a major global public health problem [2, 4, 5]. In the world, approximately 37 million people were living with HIV/AIDS in 2017 [6]. More than two-thirds (67%) of people were living with AIDS, and close to three-fourths (75%) of all AIDS-related mortalities happened in Sub-Saharan Africa (SSA) countries [6]. In 2010, SSA was one of the most affected parts of the world, with an estimated 22.9 million people living with HIV and 1.2 million deaths associated with AIDS among children and adults [2]. HIV has emerged as one of the leading causes of child mortality and morbidity in SSA countries, including Ethiopia. In SSA, approximately 1.5 million people overall are living with HIV, which has become the leading cause of mortality among children under 15 years of age, accounting for about 12.7% of all children who died of AIDS-related illnesses in 2013 [7].

Ethiopia is one of the SSA countries most affected by HIV/AIDS in all its manifestations [8]. There were an estimated 793,700 people living with HIV in 2013, including 200,300 children [7]. The Cluster of Difference 4 (CD4) cell count is one of the most important markers of HIV disease progression and a strong predictor of health status in HIV-infected children, similar to the plasma viral load [3, 7, 9].

Treatment of HIV-infected children with ART leads to immune cell reconstitution, as shown by the increase in CD4 cell counts, decreased risk of opportunistic infection, and improved survival [7]. Moreover, evidence has indicated that sufficient CD4 cell count in most patients with ART is considered when there is a rise in the range of 50–150 cells/mm3 per year with an accelerated response in the first 3 months of treatment before a stable state level is reached [10].

There are many factors that anti-retrieval therapy will affect that act as related predictors of changes in CD4 cell count. Investigations were conducted in Ethiopia to find predictors of changes in CD4 cell count among children receiving ART [4, 11–13]. These studies were conducted utilizing multiple regression and logistic regression, which are both common regression models. The repeated or longitudinal CD4 cell measurements were not included in the standard multiple regression or logistic regression model; only the cross-sectional CD4 cell data were. However, the longitudinal or linked character of the dataset restricts its utility in many real-world applications due to its underlying presumption that each individual observation must be independent of the others. This means that the results of a simple multiple regression or logistic regression model may not be useful for drawing conclusions about standard errors, parameter estimates, tests, and confidence intervals. Strong hierarchies generally result from such nested organizations since there is a significant disparity between people on average compared to circumstance. The study's patients' heterogeneity may be the cause of the discrepancy in the potential risk variables for a change in CD4 count. We suggested a linear mixed model analysis for children's data [14–16] to close this gap.

For the analysis of patient responses to therapy and clinical decision-making in resource-constrained locations, it is strongly advised to monitor clinical and diagnostic progression as well as analyze CD4 cell counts of patients on ART over time follow-up [10]. However, there is little evidence that ART follow-up in Ethiopia is improving CD4 cell counts and related variables in children. After the start of ART, the variation in CD4 cell counts between patients has remained largely uncharacterized, and the majority of published studies have failed to identify the factors that might contribute to this variation with prolonged ART usage in the nation.

Therefore, the objective of this analysis was to identify the factors that were predictive of CD4 cell count changes over time in children who began ART at Mekelle General Hospital in northern Ethiopia.

## Materials and methods

### Study design and period

An institutional-based retrospective follow-up study design was carried out among HIV-infected children (1–14 years of age) who started ART follow-up over time from 2014–2016.

### Study setting

The study was carried out in Mekelle General Hospital, Tigray Regional State, northern Ethiopia. Mekelle is the capital city of the Tigray national Regional State, which is 783 km away from Addis Ababa, the capital of Ethiopia. Besides, Mekelle General Hospital has initiated ART service delivery since September 2004, and until now, more than 10,000 HIV-infected participants have been followed-up over time with their ART services.

### Study population

The study population included HIV-positive children (1–14 years) who initiated anti-retrieval treatment in the Mekelle general hospital ART clinic.

### Inclusion criteria and exclusion criteria

Patients whose age was 1–14 years old who are attending a minimum of two visits of ART treatment in the ART clinic for refilling their prescription and who were started on ART from January 1, 2014, to January 30, 2016 at Mekelle General Hospital would be included in the study. However, patients who initiated ART and whose information is incomplete, unreadable, or their manual record is lost, as well as patients who have not had at least two follow-up CD4 cell count measures, were excluded from the study.

### Sample size and sampling procedure

All HIV-positive children who started ART follow-up over time in the General Hospital from January 1, 2014, to January 30, 2016 were included in the study. In this study, 437 HIV-infected children who had two or more CD4 cell count measurements were used in the linear mixed model analysis. Using the pediatric ART registration book, we have created a sampling frame for the general hospital using the list of all children on ART follow-up and aged 1–14 years in order to select the samples using a systematic random sampling procedure.

### Data collection tools and procedures

Data were extracted using a structured checklist built according to it, which was developed and adapted from studying different relevant literature [4, 11]. A few questionnaires, including language clarity and information, were revised, and the questionnaire was finalized for the study. The questionnaire includes socio-demographic and clinical predictor factors for children and participants and changes in CD4 cell counts over time.

### Data quality assurance and control

Four health professional nurses working at the ART clinic were employed as data collectors and extracted the dataset after they had been trained for 2 days. The data collection process was supervised by two supervisors. The data processing was carried out in private rooms. The collected data set was checked for completeness and consistency and corrected daily by the supervisors and the principal investigator.

### Variable of the study

#### Response variable

CD4 cell count measurements were the response variable for this study.

#### Independent variables

The independent variables were selected based on a review of previous literature [1, 7, 17]. The independent variables that were used in this study are shown in Table 1 with their respective categories.

**Table 1:** Independent variables

Variables	Categorizations of independent variables
WHO clinical stage	Baseline WHO clinical stage level of children (stage-I, stage-II, stage-III, stage-IV)
Sex	Sex of child (male, female)
Co-infection HIV/TB	Co-infection HIV with TB (yes, no)
Education level	Education level of child (elementary and below, secondary and above)
Residence	Residence place (urban, rural)
Functional status	Baseline functional status (working, ambulatory, bedridden)
Opportunistic infection	Opportunistic infection at ART start (yes, no)
Adverse drug events	Adverse drug events (yes, no)
Age category	Age category of child (< 2 years, 2–5 years, 6–14 years)
Adherence level	Adherence level of medication (good, fair, poor)
Time	Follow up time points at which CD4 cells count was recorded (in months)

#### Operational definition of variables

Base line data: refers to the data before antiretroviral therapy started [8].

##### Co-infection HIV/TB

Refers to patients living with HIV and also developing coexistent TB infection [2].

Good Adherence level to medications: Children living with HIV/AIDS on antiretroviral therapy recorded to have taken 95% or higher of their prescribed antiretroviral therapy medication or missed <  = 3 doses as to their agreement with health care provider [8, 11].

Poor adherence level to medications: level of adherence below 95% of their prescribed antiretroviral therapy medication or missed > 3 doses as to their agreement with health care provider [8, 11].

##### Opportunistic infections

HIV-infected child on antiretroviral therapy developed one or more recorded history of opportunistic infections within follow up time.

Advanced WHO clinical stage: are clinical stage III and IV baseline stages of HIV-infected children throughout registration to antiretroviral therapy [13].

##### Mild WHO clinical stages

Are clinical stage I and II baseline clinical stages of HIV-infected children within antiretroviral therapy registration [13].

##### CD4 cell count change

Refers to the CD4 cell count change within follow-up time of patients.

### Data processing and statistical analysis

Data were extracted, entered, cleaned, decoded, and analyzed using the statistical software R version 3.6.3. Descriptive statistics such as frequency, percentages, and figures were used to describe children’s characteristics. A square root transformation was implemented to get rid of skewness in the CD4 cell count data set, and all analyses were performed using the transformed result data. Moreover, to determine the model that best fits the data set, exploratory data analysis was employed first, especially by assessing individual CD4 cell counts over time using individual profile plots and average profile plots. Therefore, individual profile plots of patients were constructed for the first 50 patients to provide a rough image of how patients changed and to provide explanations for variation within and between subjects. Similarly, the average profile plots were constructed to explain the overall mean shift in CD4 cell count for HIV-infected children. To assess the predictors associated with CD4 cell count changes, all predictors were considered in the multivariable linear mixed regression model analysis with a random effect. Variables with a *p*-value < 0.05 were found to be statistically significant in a multivariable random S-I of LMM analysis. Moreover, the maximum likelihood parameter estimation approach was used.

### Selection of covariance structure

The most common types of covariance structures in repeated measures are unstructured, independent, compound symmetry, and identity, and the magnitude of residual errors was also considered in model selection. The covariance structure model was employed to determine the predictors associated with CD4 cell count over time. As a result, a model with the least within-individual variation when compared to other models’ residual variability was selected. Moreover, these covariance structures will reduce the probability of model misspecification [18].

### Linear mixed effects model analysis

In this study, these longitudinal models were fitted based on either of the following three model mechanisms [14–16].

I. **Random slopes and random intercepts model**

This model analysis used both random intercepts and random slopes, in which both random intercepts and slopes are allowed to vary. So, the scores on the outcome variable for each repeated measurement are predicted by the random intercepts and random slopes that vary across individual patients. These models were performed to measure covariates and predict the random effect at the same time. Besides, we identify the variations explained by within-subjects and between-subjects random intercept and random slope model analysis. The general structure for LMM is expressed as;

1 $${{\varvec{y}}}_{{\varvec{i}}{\varvec{j}}}=\frac{{{\varvec{\beta}}}_{0}{ + {{\varvec{\beta}}}_{1*}{\varvec{X}}}_{{\varvec{i}}}{\varvec{j}}}{{\varvec{f}}{\varvec{i}}{\varvec{x}}{\varvec{e}}{\varvec{d}}\,{\varvec{e}}{\varvec{f}}{\varvec{f}}{\varvec{e}}{\varvec{c}}{\varvec{t}}{\varvec{s}}}+{\frac{{{\varvec{b}}}_{{\varvec{i}},0}+ {{\varvec{b}}}_{{\varvec{i}},1*}{\varvec{z}}{\varvec{i}} }{{\varvec{r}}{\varvec{a}}{\varvec{n}}{\varvec{d}}{\varvec{o}}{\varvec{m}}\,{\varvec{e}}{\varvec{f}}{\varvec{f}}{\varvec{e}}{\varvec{c}}{\varvec{t}}{\varvec{s}}}}_{.}+\frac{{\in }_{{\varvec{i}}}}{{\varvec{r}}{\varvec{a}}{\varvec{n}}{\varvec{d}}{\varvec{o}}{\varvec{m}}\,{\varvec{e}}{\varvec{r}}{\varvec{r}}{\varvec{o}}{\varvec{r}}},$$

$$\mathbf{i}=1, \dots \dots \dots \dots \mathbf{m} \mathrm{and} \mathbf{j}=1,\boldsymbol{ }2,\boldsymbol{ }\dots \dots {{\varvec{n}}}_{{\varvec{i}}}.$$

where ${{\varvec{y}}}_{{\varvec{i}}}=({{\varvec{y}}}_{{\varvec{i}}1}, {{\varvec{y}}}_{{\varvec{i}}2}, {{\varvec{y}}}_{{\varvec{i}}3} , \dots \dots .. {{\varvec{y}}}_{{\varvec{i}}{\varvec{n}}})$.^T^ is the dependent variable,${X}_{ij}$ is the vector of indicator variables for the study predictors, ${{\varvec{b}}}_{{\varvec{i}}}\sim {{\varvec{N}}}_{{\varvec{q}}}(0,\boldsymbol{\varphi })$, ${{\varvec{\varepsilon}}}_{{\varvec{i}}}\sim {{\varvec{N}}}_{{\varvec{n}}{\varvec{i}}}$(0,${{\varvec{\delta}}}^{2}{\varvec{I}}$)

${\varvec{\beta}}=$ Fixed effects, ${{\varvec{b}}}_{{\varvec{i}}}$= Random effect for unit $i$, $\boldsymbol{\varphi }=$ Between-subjects covariance matrix, ${{\varvec{\delta}}}^{2}{\varvec{I}}$ = Within-subjects covariance matrix, ${{\varvec{b}}}_{{\varvec{i}}}$ and ${\in }_{{\varvec{i}}}$ is assumed to be independent. ${X}_{i}$ is an ${n}_{i}*j$ matrix with ${j}^{th}$ column, matrix ${Z}_{i}$ is an ${n}_{i}*k$ matrix. Both ${X}_{i}$ and ${Z}_{i}$ depend on $i$ through ${t}_{i}$.

Now, the Intra-Class Correlation coefficient (ICC) is defined as a set of coefficients representing the relationship between variables of the same individuals that decompose into two independent components. Thus, the ICC explained by the individuals in the population is given by this formula:

2 $$\mathrm{ICC}-\mathrm{CD}4\left(\right)=\frac{\mathbf{V}\mathbf{a}\mathbf{r}\mathbf{i}\mathbf{a}\mathbf{n}\mathbf{c}\mathbf{e} \,\mathbf{b}\mathbf{e}\mathbf{t}\mathbf{w}\mathbf{e}\mathbf{e}\mathbf{n} [\mathrm{Var}\left(\mathrm{bi}\right)]}{\mathbf{V}\mathbf{a}\mathbf{r}\mathbf{i}\mathbf{a}\mathbf{n}\mathbf{c}\mathbf{e} \,\mathbf{b}\mathbf{e}\mathbf{t}\mathbf{w}\mathbf{e}\mathbf{e}\mathbf{n} \left[\mathrm{Var}\left(\mathrm{bi}\right)\right]+ \mathbf{V}\mathbf{a}\mathbf{r}\mathbf{i}\mathbf{a}\mathbf{n}\mathbf{c}\mathbf{e}\, \mathbf{w}\mathbf{i}\mathbf{t}\mathbf{h}\mathbf{i}\mathbf{n} [(\mathrm{Var}\left(\mathrm{ei}\right)] }$$

Where, e_i_ is the error terms and b_i_ is a random intercept are assumed to be mutually independent and ICC_measures_ = 1 − ICC_individual_ [1, 16].

II. **Random intercept only model**

This is the simplest example of hierarchical model analysis, in which intercepts are allowed to vary and there are no predictor variables at all. It has only an intercept term and variances at the measurement and individual levels. Since the model doesn’t contain a random slope, the true individual change is a horizontal line with y-intercept b_0_.

The model can be expressed as: -

3 $${{\varvec{y}}}_{{\varvec{i}}{\varvec{j}}}=\frac{{{\varvec{\beta}}}_{0}{ + {{\varvec{\beta}}}_{1*}{\varvec{X}}}_{{\varvec{i}}}{\varvec{j}}}{{\varvec{f}}{\varvec{i}}{\varvec{x}}{\varvec{e}}{\varvec{d}}\,{\varvec{e}}{\varvec{f}}{\varvec{f}}{\varvec{e}}{\varvec{c}}{\varvec{t}}{\varvec{s}}}+{\frac{ {{\varvec{b}}}_{{\varvec{i}},0} }{{\varvec{r}}{\varvec{a}}{\varvec{n}}{\varvec{d}}{\varvec{o}}{\varvec{m}}\,{\varvec{e}}{\varvec{f}}{\varvec{f}}{\varvec{e}}{\varvec{c}}{\varvec{t}}{\varvec{s}}}}_{,}+\frac{ {\in }_{{\varvec{i}}{\varvec{j}}} }{{\varvec{r}}{\varvec{a}}{\varvec{n}}{\varvec{d}}{\varvec{o}}{\varvec{m}}\,{\varvec{e}}{\varvec{r}}{\varvec{r}}{\varvec{o}}{\varvec{r}}}, \mathbf{i}= 1, \dots \dots \dots \mathbf{m}$$

${{\varvec{\beta}}}_{0}$ Is the intercept of fixed effect that is a constant over time, *b*_0*i*_ is the random effect representing between-subjects variation, ${\in }_{ij}$ is the error [16].

III. **Random slope model**

A random slopes model is a longitudinal model analysis in which slopes are allowed to vary. Thus, the scores on the response variable for each repeated measurement are predicted by the slope that varies between patients and subjects. The previous models are sometimes called unconditional (intercept only) models because there are no measured covariates to predict the random effect. Now, when occasions vary, we have different sets of measurements taken at different points over time for different individuals [14–16]. Such models are often interested in assessing how a longitudinal outcome variable is associated with a covariate whose value changes over time. Such covariates are called time-varying covariates Xi.

4 $${{\varvec{y}}}_{{\varvec{i}}{\varvec{j}}}=\frac{{{\varvec{\beta}}}_{0}{ + {{\varvec{\beta}}}_{1*}{\varvec{X}}}_{{\varvec{i}}}{\varvec{j}}}{{\varvec{f}}{\varvec{i}}{\varvec{x}}{\varvec{e}}{\varvec{d}}\,{\varvec{e}}{\varvec{f}}{\varvec{f}}{\varvec{e}}{\varvec{c}}{\varvec{t}}{\varvec{s}}}+{\frac{ {{\varvec{b}}}_{{\varvec{i}},1*}{\varvec{z}}{\varvec{i}} }{{\varvec{r}}{\varvec{a}}{\varvec{n}}{\varvec{d}}{\varvec{o}}{\varvec{m}}\,{\varvec{e}}{\varvec{f}}{\varvec{f}}{\varvec{e}}{\varvec{c}}{\varvec{t}}{\varvec{s}}}}_{,}+\frac{ {\in }_{{\varvec{i}}} }{{\varvec{r}}{\varvec{a}}{\varvec{n}}{\varvec{d}}{\varvec{o}}{\varvec{m}}\,{\varvec{e}}{\varvec{r}}{\varvec{r}}{\varvec{o}}{\varvec{r}}{\varvec{s}}}, \mathbf{i}= 1, \dots \dots \dots \mathbf{m}$$

### Model selection and comparison

In order to select the best and final model that appropriately fits the given longitudinal data set, it is essential to compare the different linear mixed models using different techniques and methods. Therefore, Akaki Information Criteria (AIC) and Bayesian Information Criteria (BIC) that are calculated from deviance based on the number of estimated parameters k are also most convenient at a 5% level of significance. Accordingly, a model with a smaller value of AIC/BIC was selected as the preferable model [16, 19, 20].

## Results

### Socio-demographic and clinical characteristics of participants

Out of a total of 437 HIV-infected children chosen for the study, 284 (64.99%) were male participants. The estimated mean change in CD4 cell count of the participants was 340.9 (95% CI: 334.4, 347.5) cells/mm^3^ for every 6 months of follow-up after ART start. On the subject of WHO clinical stages, 16.7%, 38.22%, 29.52%, and 15.56% of HIV-infected children were in stages I, II, III, and IV, respectively. Of the children’s patients, 204 (46.68%) were at a good adherence level; 291 (66.59%) had working functional status; and 160 (36.61%) had opportunistic infections during ART start. Regarding co-infection with HIV and TB, 33.64% of HIV-infected children were TB positive (see Table 2).

**Table 2:** Socio-demographic and clinical characteristic of patients who were on ART in Mekelle general hospital, from 2014–2016 (*n* = 437)

Variable	Category	Frequency	Percentage
WHO clinical stage	Stage-I	73	16.7
	Stage-II	167	38.22
	Stage-III	129	29.52
	Stage-IV	68	15.56
Sex	Male	284	64.99
	Female	153	35.01
Co-infection HIV/TB	No	292	66.82
	Yes	147	33.64
Education level	Elementary and below	143	32.72
	Secondary and above	294	67.28
Residence	Urban	286	65.45
	Rural	151	34.55
Functional status	Working	291	66.59
	Ambulatory	98	22.43
	Bedridden	48	10.98
Opportunistic infection	No	160	36.61
	Yes	277	63.39
Adverse drug events	No	269	61.56
	Yes	168	38.44
Age category	< 2 years	43	9.84
	2–5 years	374	85.58
	6–14 years	20	4.577
Adherence level	Good	204	46.68
	Fair	135	30.89
	Poor	98	22.43

### Exploratory data analysis of changes in $\sqrt{\mathbf{C}\mathbf{D}4}$ cell count after starting ART

#### Individual profile plot of progression curve analysis

**Fig. 1:**
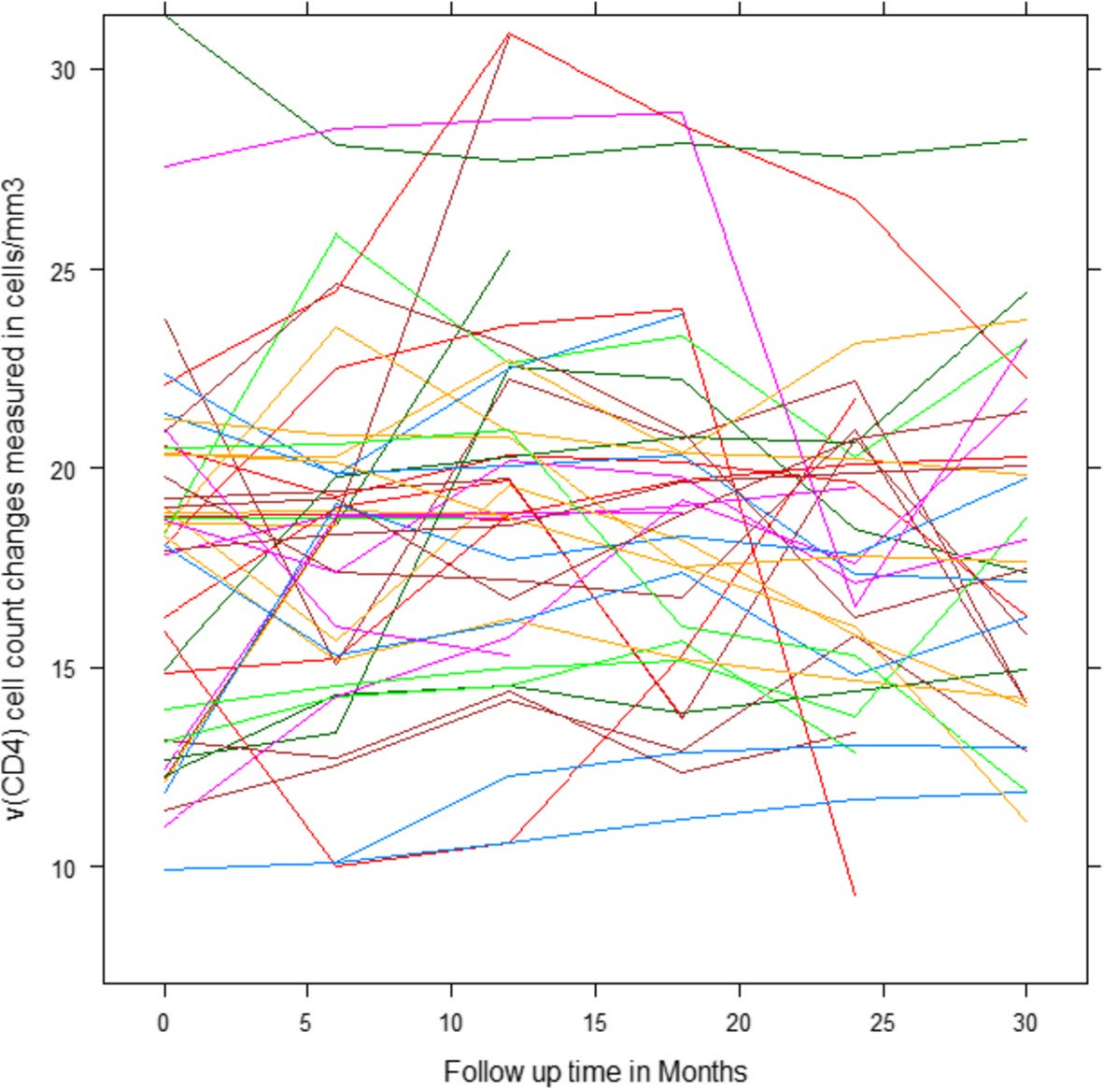
Individual profile plots in $\sqrt{CD4}$ cell count of the first 50 ART followers over time

The visualized pattern of $\sqrt{CD4}$ cell count measurements of the patient’s overtime and the first 50 individual profile plots of subjects were considered. This figure indicated that the variability within and between patients had a slightly decreasing trend for each respondent throughout the follow-up over time. For responses, the majority (but not all) of observations were slightly turned down throughout the follow-up period. Therefore, the variation within and between the patients throughout the time of follow-up decreased each response from a visit time to a visit time (see Fig. 1).

### Mean profile plot stratified by co-infection HIV/TB

**Fig. 2:**
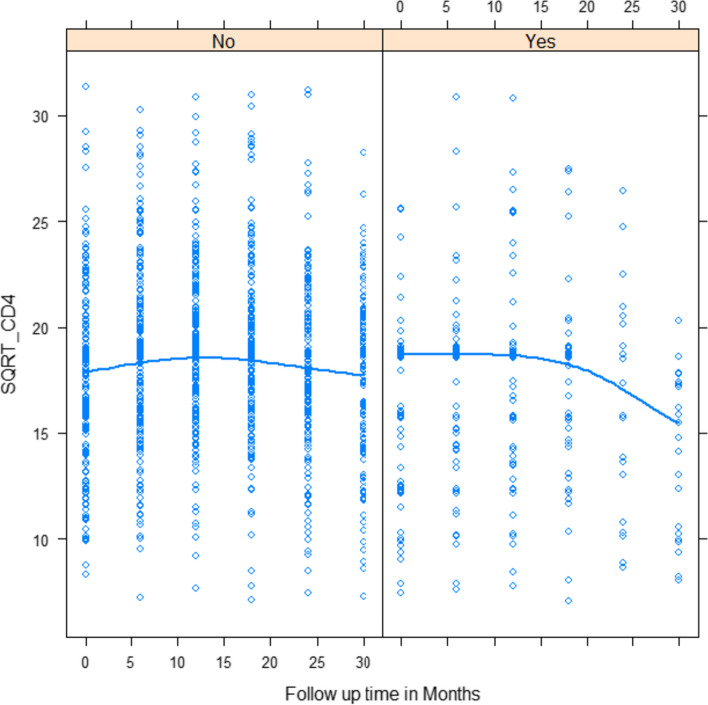
Smoothing mean profile in $\sqrt{CD4}$ cell on ART follow-up stratified by co-infection HIV/TB

To sum up, the overall smooth average profile plot in $\sqrt{CD4}$ cell count changes on the first 50 subjects showed signs of variation within and between patients in both categories. The patients had more variation in $\sqrt{CD4}$ cell values at the end and adjustments over time at the beginning of ART. Besides, the average change in $\sqrt{CD4}$ cell counts over time for those who were co-infected with TB indicated that they remained constant in the first 5 months, followed by a modest decrease of up to 10 months. In the end, it was a dramatic decrease from the 10^th^ to the last month. It is evident that the total mean $\sqrt{CD4}$ cell count decreased over time and remained constant over time (see Fig. 2).

**Fig. 3:**
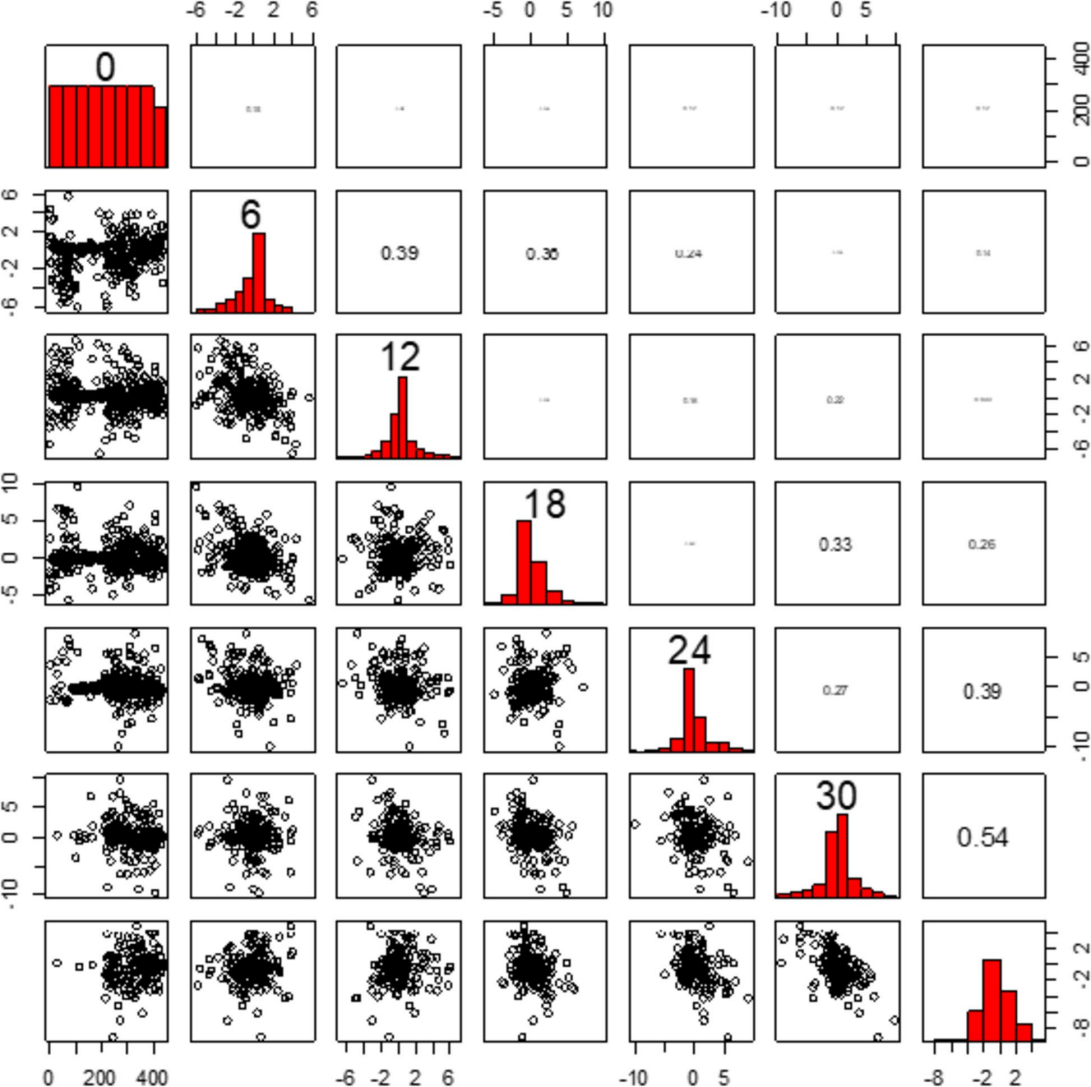
Exploring correlation structures on individual heterogeneity of HIV-infected children

The upper triangle Fig. 3 shows that the correlation structure values depend on the individual heterogeneity of patients. This indicates that the time progression and strength of association with the change in CD4 count are decreasing. Thus, as time gets closer, we have a higher correlation over time. That means, as time moves on, the correlation is reducing. Therefore, correlation gets weaker over time because CD4 count depends on time (see Fig. 3).

### Predictors associated with $\sqrt{\mathbf{C}\mathbf{D}4}$ cell count changes over time

#### Model and covariance structure selection

The study used the AIC, BIC, and Log-lik criterion to compare different models. For each model, the value is computed as AIC =  − 2*ln(likelihood) + 2(p + k) and BIC = -2*ln(likelihood) + ln(N)*k. where, k is the number of parameters estimated and N is the number of observations.

Based on the following statistical values of the AIC, BIC, and Log-Lik criteria, a linear mixed effect model with a random S-I model was preferable for modelling since the lowest value is preferable. In this study, the modeling analysis of a linear mixed model with random S-I was assumed to compensate for individual variations in $\sqrt{CD4}$ cell count at baseline and over time, respectively. The adequacy of the model fitted could also be affected by the error covariance structure selected. This is because the distribution of errors requires that an error covariance structure be employed. As a result, four different variance–covariance structures were compared for each candidate model. Moreover, the random and fixed effects were calculated by comparing various covariance structures. Because the unstructured structure gave the lowest information criterion in all possible combinations, it was selected to determine the random effect (see S1 and S2 Tables).

After adjusting for possible confounding predictors using the multivariable LMM with random slope and random intercept analysis, it was found that covariates such as WHO clinical stage, co-infection HIV/TB, functional status, opportunistic infections, and age were significantly associated with $\sqrt{CD4}$ cell count changes over time. While sex, education level, residence, adverse drug events, adherence level, and time of follow-up did not significantly affect in $\sqrt{CD4}$ cell count changes over time.

In children who are HIV-positive, an increase in the predictor variable causes an increase in CD4 cell count, while an increase in the independent variable causes a decrease in CD4 cell count, as shown by the positive (+ ve) sign of the assessed value of parameters ( value in Table 3). The category with the positive sign of estimate had a higher CD4 cell count than its counterpart for categorical variables like opportunistic infection patients and TB co-infected patients, and the category with the negative sign of assessment had a lower CD4 cell count than its counterpart.

**Table 3:** Multivariable LMM analyses of predictors associated with change of CD4 cell counts overtime who were on ART follow-up among HIV-infected children in Mekelle general hospital (2014–2016)

**Covariate**	**Coefficient**	**(95% CI)**		***P*-value**
		Lower	Upper	
Intercept	19.1	17.9	20.18	0.000*
Time	0.022	-0.005	0.048	0.113
WHO clinical stage (reff. = stage-I)	1.00			
Stage-II	-1.088	-2.20	0.022	0.056
Stage-III	-0.59	-1.91	0.732	0.385
Stage-IV	-1.30	-2.37	-0.23	0.018*
Sex (reff. = male)	1.00			
Female	-0.46	-1.16	0.241	0.200
Co-infection HIV/TB (reff. = no)	1.00			
Yes	-1.78	-2.58	-0.98	0.000*
Education level (reff. = secondary and above)	1.00			
Elementary and below	-0.56	-1.29	0.163	0.130
Residence (reff. = urban)	1.00			
Rural	0.079	-0.55	0.708	0.808
Functional status (reff. = ambulatory)	1.00			
Working	0.602	-0.151	1.357	0.119
Bedridden	-1.74	-2.81	-0.68	0.002*
Opportunistic infection (reff. = yes)	1.00			
No	1.33	0.51	2.14	0.002*
Adverse drug events (reff. = no)	1.00			
Yes	0.068	-0.99	1.12	0.899
Age category (reff. = < 2 years)	1.00			
2–5 years	-0.43	-0.82	-0.04	0.033*
6–14 years	-1.02	-1.47	-0.56	0.000*
Adherence level (reff. = good)	1.00			
Fair	0.211	-0.75	1.17	0.667
Poor	0.115	-0.52	0.75	0.725

*Reff* Reference for category variables

N.B: LR test: chi2(3) = 618.0, *p*-value = 0.0000

Wald: chi2 (15) = 106.2, *p*-value = 0.0000

^*^Significant at 5% level of significance

Children in bedridden functional status have lower $\sqrt{CD4}$ cell counts compared to ambulatory children. The mean change in $\sqrt{CD4}$ cell count for bedridden patients was 1.74 (β = -1.74, 95% CI: -2.81, -0.68) times lower compared to ambulatory patients, controlling for the other variable.

Similarly, the average change in $\sqrt{CD4}$ cell count for HIV-infected children who lived in TB was about 1.78 (β = -1.78, 95% CI: -2.58, -0.98) times lower than that for those patients who were negative.

Patients who started ART at WHO clinical stage IV reported smaller increases in CD4 cell counts relative to those who started ART at the stage I. Besides, the mean change of the CD4 cell count for patients with WHO clinical stage IV was 1.30 (β = -1.30, 95% CI: -2.37, -0.23) times lower than that of those patients who with WHO clinical stage I.

Age was closely associated with CD4 cell count changes, suggesting that the average $\sqrt{CD4}$ cell count of children ages 2–5 and 6–14 years was 0.43 (β = -0.43; 95% Cl: -0.82, -0.04) and 1.02 (β = -1.02; 95% Cl: -1.47, -0.56) times significantly lower than the reference group, respectively. At baseline, the mean $\sqrt{CD4}$ cell count among children who hadn’t had an opportunistic infection was 1.33 (β = 1.33, 95% CI: 0.51, 2.14) times higher than the mean $\sqrt{CD4}$ cell count among children who had an opportunistic infection (see Table 3).

The standard deviation estimated values for the random slope and intercept were found to be 0.13 and 2.98, respectively. This indicates that there is substantial variability in random slopes and intercepts between and within patients and that the mean change in $\sqrt{CD4}$ cell count varies across HIV-infected children.

Moreover, Table S2 results show that ICC gives strong correlation evidence that variability existed between the HIV patients. Therefore, the ICC of this study was: $\uprho =\frac{8.86}{ 8.86+ 5.6 }$=$\frac{8.86}{ 14.45 }$  = 0.613. Therefore, 61.3% of the explained variation in $\sqrt{CD4}$ cell count existed between patients, and the remaining 38.7% of variation existing within patients.

## Discussion

Patients with HIV are now living longer and dying less due to highly active ART. Effective ART had a major impact on people with HIV infection's longevity and ability to avoid opportunistic infections. According to research from the past and the current study, ART is a useful treatment for HIV that can reduce viral load to undetectable levels.

The aim of this study was to identify the rate of CD4 cell count change over time and determine its associated predictors among HIV-infected children who started antiretroviral therapy. The individual profile plots indicated the presence of variation in $\sqrt{CD4}$ cell counts between and within subjects. The separated mean profile plot also showed that, on average, $\sqrt{CD4}$ cell counts appeared to change rapidly over time on ART follow-up.

The results of LMM for random effects indicated that there was a significant variation in the change in $\sqrt{CD4}$ cell count across the patients. In the multivariable LMM with variability between and within patients accounted for, about 61.3% and 38.7% of the variations were observed for the change in $\sqrt{CD4}$ cell count, respectively. It was in agreement with a study in Ethiopia [1]. As a result, the degree of suppression of viral replication increases, even though it should be recognized that such plots are mean plots that may be different from individual profile plots, which may show that certain HIV-infected children respond better than others.

According to this study, children's age and CD4 cell count changes had negative associations. The CD4 cell count square root odds were 0.43 and 1.02 times lower than the reference group. This finding is consistent with those of studies carried out in northwest Ethiopia [10, 21]. It is well known that as age increases, thymic activity lowers, which will reduce CD4 cell counts as the thymus is the primary site of CD4 cell count progression [10].

Children with opportunistic infections had major negative impacts on CD4 cell count changes over time. This indicated that children with no opportunistic infections had higher $\sqrt{CD4}$ cell counts over time. This is consistent with studies done in northwest Ethiopia [10, 22]. The possible reason may be that opportunistic infections recover HIV pathogenesis and further reinforce the value of prophylaxis.

Children with advanced HIV stages have poorer immunological recovery than those with early HIV stages because opportunistic infections are more common in patients with advanced HIV stages [4].

The present CD4 cell count of patients who had opportunistic infections was lower than that of their counterparts. This study is in line with a study conducted in Ethiopia [4, 23]. This may be because a reduction in CD4 cell counts leads to cellular and, consequently, humeral deficiencies.

Co-infection HIV and TB are also highly associated with a decrease in CD4 cell count over time. The finding is consistent with previous studies in Ethiopia [2, 19], which indicated that patients with TB comorbidity are associated with decreased $\sqrt{CD4}$ cell counts, reduced immune repair, and reduced survival, resulting in faster disease progression.

Functional status since the initiation of ART in patients was also found to be a statistically significant predictor of CD4 cell count change over time. Therefore, bedridden patients have a low rate of recovery of CD4 cell count changes. Otherwise, children patients who are in working functional status can take the prescribed medication by themselves at the time given by the health professionals, which leads to a good recovery of CD4 cell count. This result is consistent with another study done in Ethiopia, which also reported a significant association between functional status and changes in CD4 cell count over time [24].

However, there are additional variables besides HIV that can affect CD4 cell numbers. Rural living [22] and lack of education [4] among patients are predictors of reduced CD4 cell count responses to anti-retrieval therapy, but not in the context of these recent investigations. The probable explanation is that educated HIV/AIDS patients and urban residents have a better awareness of the disease status and are more likely to understand instructions on medicine consumption than rural residents.

Finally, the shift in CD4 cell count was significantly impacted negatively by WHO clinical stage-IV. As a result, when compared to patients in stage I, the CD4 squared root chances for patients in stage IV reduced by 1.30. This conclusion is in line with research done in Ethiopia [17, 25].

### Strengths and limitation of the study

To account for the patients with/between variations in children, this study used the most appropriate statistical linear mixed model analysis (longitudinal model technique). Additionally, this research will help policymakers create a better plan for managing HIV-positive people. Additionally, the investigation will serve as a starting point for future scholars. The final drawback of this study is that, due to the retrospective follow-up nature of the results, which did not include all the crucial predictor variables like viral load, additional clinical parameters due to a lack of materials, or technical issues, the causal association cannot be precisely well-defined. Additionally, the impact of CD4 cell count can differ due to a lack of high-quality and accessible datasets. These could be regarded as study gaps.

## Conclusions

This study found that patients receiving ART experienced a significant change in CD4 cells over time. Because 61.3% of the variation in CD4 cells explained between patients and the remaining 38.7% within patients, such nested data structures are often strong correlation evidence. Co-infection of HIV/TB, functional status, age category of children, WHO clinical stage, and opportunistic infections are potential predictors of CD4 cells count change.

Therefore, special guidance and attention is also required, especially for those patients who have an opportunistic infections, higher WHO clinical stages, co-infections with HIV and TB, and bedridden functional status.

### Supplementary Information

**Additional file 1: S1 Table.** Selection of correlation structure for ART data.** S2 Table.** Random parameter estimates and random effects models comparisons with smallest AIC, BIC, and LogLik for ART data.** Appendix.** R-commands for data analysis.

## Data Availability

Data will be available upon request from the corresponding author.

## Abbreviations

AIDS: Acquired Immune Deficiency Syndrome
AIC: Akaike Information Criteria
ART: Anti-Retroviral Therapy
BIC: Bayesian Information Criteria
CD4: Cluster of Difference 4
HIV: Human Immunodeficiency Virus
ICC: Intra-Class Correlation
LMM: Linear Mixed Model
S-I: Random Slope and Intercept
SSA: Sub–Saharan Africa
TB: Tuberculosis
WHO: World Health Organization
