# Supplementary material for: Predictors associated with CD4 cell count changes over time among HIV-infected children on anti-retroviral therapy follow-up in Mekelle General Hospital, Northern Ethiopia, 2019: a retrospective longitudinal study
Source: BMC Pediatr. 2023 Dec 12;23:628. doi: 10.1186/s12887-023-04401-7 (PMC10714531; doi:10.1186/s12887-023-04401-7)
Supplement: Supplementary file 1 — Additional file 1: S1 Table. Selection of correlation structure for ART data. S2 Table. Random parameter estimates and random effects models comparisons with smallest AIC, BIC, and LogLik for ART data. Appendix. R-commands for data analysis. [file 12887_2023_4401_MOESM1_ESM.docx]

**Supplementary information**

**Additional file 1:** S1 Table. Selection of correlation structure for ART data.

|  | Covariance structures | | | |
| --- | --- | --- | --- | --- |
| Information Criteria | Independent | UN | CS | Identity |
| AIC | 9960.5 | 9907.6** | 10294.2 | 10310.8 |
| BIC | 10066.3 | 10018.8** | 10399.9 | 10410.9 |
| Log-lik | -4961.3 | -4933.8** | -5128.1 | -5137.3 |
| UN: Unstructured, CS: Compound Symmetry, ** means**: “smallest” implying best model. | | | | |

**Additional file 2:** S2 Table. Random parameter estimates and random effects models comparisons with smallest AIC, BIC, and LogLik for ART data.

|  |  |  | Information Criteria (Goodness-of-fit) | | |
| --- | --- | --- | --- | --- | --- |
| Random-effects Parameters | Estimate | stdDev | AIC | BIC | Log-lik |
| id: Unstructured |  |  |  |  |  |
| Random slope and intercept (S–I) | Β_1_ | 0.13 | 9873.5** | 9990.4** | -4915.8** |
|  | Β_0_ | 2.98 |  |  |  |
|  | _b_ | -0.56 |  |  |  |
|  | e | 2.37** |  |  |  |
| Random slope | Β_1_ | 0.12 | 10288.3 | 10394 | -5125.2 |
|  | e | 3.15 |  |  |  |
| Random intercept | Β_0_ | 2.41 | 9981.4 | 10086.98 | -4971.7 |
|  | e | 2.70 |  |  |  |
| AIC: Akaike Information Criteria, BIC: Bayesian Information Criteria, LogLik: Log-Likelihood, _b_: Cor (Intercept, time), Β_1_: Time, Β_0_: Intercept, e: Residual, ** means**: “smallest” implying best model. | | | | | |

**Appendix**

**R-commands for data analysis**

### Changing Variables as.integer, as.numeric, as.factor, as.character etc..###

> mydata$id=as.factor(mydata$id)

> mydata$time=as.integer(mydata$time)

> mydata$CD4=as.numeric(mydata$CD4)

> mydata$WHO_stage=as.factor(mydata$WHO_stage)

> mydata$SQRT_CD4=as.numeric(mydata$ SQRT_CD4)

> mydata$sex=as.factor(mydata$sex)

> mydata$HIV_TB=as.factor(mydata$HIV_TB)

> mydata$education=as.factor(mydata$education)

> mydata$function_status=as.factor(mydata$function_status)

> mydata$residence=as.factor(mydata$residence)

> mydata$Opportunistic_infection=as.factor(mydata$Opportunistic_infection)

> mydata$Adverse.drug.events=as.factor(mydata$Adverse.drug.events)

> mydata$Age.category=as.factor(mydata$Age.category)

> mydata$Adherence.status=as.o

## Giving "References" For Our Categorical Variables in Your Data Analysis ##

> library(stats)

> mydata$WHO_stage=relevel(mydata$WHO_stage,ref="stage-I")

> mydata$sex=relevel(mydata$sex,ref="male")

> mydata$HIV_TB=relevel(mydata$HIV_TB,ref="NO")

> mydata$function_status=relevel(mydata$function_status,ref="ambulatory")

> mydata$education=relevel(mydata$education,ref="Secondary and above")

> mydata$residence=relevel(mydata$residence,ref="urban")

> mydata$Opportunistic_infection=relevel(mydata$Opportunistic_infection,ref="yes")

> mydata$Adverse.drug.events=relevel(mydata$Adverse.drug.events,ref="no")

> mydata$Age.category=relevel(mydata$Age.category,ref="2-5 years")

> mydata$Adherence.status=relevel(mydata$Adherence.status,ref="good")

# Fit Multi-variable LMM of RI-RS on Covariate's with ML estimation techniques ##

library(nlme)

> fitlmm_1<-lme(SQRT_CD4~time + WHO_stage + sex + HIV_TB + function_status+education+residence+Opportunistic_infection+Age.category+Adverse.drug.events+Adherence.status,

+ random = ~time + 1 |id, method="ML",data =mydata)

> summary(fitlmm_1)

## The overall smooth average of √(CD4) cell count progression stratified by co-infection HIV/TB#

> library(lattice)

> xyplot(SQRT_CD4~time|HIV_TB,group=id,data=mydata[mydata$id %in% sample(mydata$id,50), ],xlab="Follow up time in Months",type="l",ylim=range(mydata$SQRT_CD4))

## Individual profile plot of √(CD4) cell count for the first 100 (n/4) sample followers of HIV positive Children patients ##

> xyplot(SQRT_CD4~time,group=id,data=mydata[mydata$id %in% sample(mydata$id,100), ],xlab="Follow up time in Months",ylab="√(CD4) cell count changes measured in cells/mm3",type="l",ylim=range(mydata$SQRT_CD4))

# Corelation Structure of changes in √(CD4) counts and progression on time ##

> library(lattice)

> library(nlme)

> fitlmm_1<-lme(SQRT_CD4~time + WHO_stage + sex + HIV_TB + function_status+education+residence+Opportunistic_infection+Age.category+Adverse.drug.events+Adherence.status,

+ random = ~time + 1 |id, method="ML",data =mydata)

> df$lmres=resid(fitlmm_1)

> df$roundyr=round(df$time)

> dfw=reshape(df[,c("id","lmres","roundyr")],direction="wide",v.names="lmres",timevar="roundyr",idvar="id")

> pairs(dfw,c(0,6,12,18,24,30))

> panel.hist=function(x, ...){

+ usr=par("usr");on.exit(par(usr))

+ par(usr=c(usr[1:2],0,1.5))

+ h=hist(x,plot = F)

+ breaks=h$breaks;

+ nB=length(breaks)

+ y=h$counts;

+ y=y/max(y)

+ rect(breaks[-nB],0,breaks[-1],y,col="red", ...)}

> panel.cor=function(x,y,digits=2,prefix="",cex.cor){

+ usr=par("usr");on.exit(par(usr))

+ par(usr=c(0,1,0,1))

+ r=abs(cor(x,y,use="pairwise.complete.obs"))

+ txt=format(c(r,0.1234),digits = digits)[1]

+ txt=paste(prefix,txt,sep="")

+ if(missing(cex.cor))cex<-0.8/strwidth(txt)

+ text(0.5,0.5,txt,cex=cex*r)

+ }

> pairs(dfw,c(0,6,12,18,24,30),upper.panel=panel.cor,diag.panel= panel.hist)
